# Development and evaluation of an instrument for the critical appraisal of randomized controlled trials of natural products

**DOI:** 10.1186/1472-6882-9-11

**Published:** 2009-04-23

**Authors:** Tannis Jurgens, Anne Marie Whelan, Melissa MacDonald, Lindsay Lord

**Affiliations:** 1 College of Pharmacy, Dalhousie University, 5968 College Street, Halifax, Nova Scotia, B3H 3J5, Canada; 2 Pharmacy Consultant, Department of Family Medicine, Dalhousie University, 5968 College Street, Halifax, Nova Scotia, B3H 3J5, Canada

## Abstract

**Background:**

The efficacy of natural products (NPs) is being evaluated using randomized controlled trials (RCTs) with increasing frequency, yet a search of the literature did not identify a widely accepted critical appraisal instrument developed specifically for use with NPs. The purpose of this project was to develop and evaluate a critical appraisal instrument that is sufficiently rigorous to be used in evaluating RCTs of conventional medicines, and also has a section specific for use with single entity NPs, including herbs and natural sourced chemicals.

**Methods:**

Three phases of the project included: 1) using experts and a Delphi process to reach consensus on a list of items essential in describing the identity of an NP; 2) compiling a list of non-NP items important for evaluating the quality of an RCT using systematic review methodology to identify published instruments and then compiling item categories that were part of a validated instrument and/or had empirical evidence to support their inclusion and 3) conducting a field test to compare the new instrument to a published instrument for usefulness in evaluating the quality of 3 RCTs of a NP and in applying results to practice.

**Results:**

Two Delphi rounds resulted in a list of 15 items essential in describing NPs. Seventeen item categories fitting inclusion criteria were identified from published instruments for conventional medicines. The new assessment instrument was assembled based on content of the two lists and the addition of a Reviewer's Conclusion section. The field test of the new instrument showed good criterion validity. Participants found it useful in translating evidence from RCTs to practice.

**Conclusion:**

A new instrument for the critical appraisal of RCTs of NPs was developed and tested. The instrument is distinct from other available assessment instruments for RCTs of NPs in its systematic development and validation. The instrument is ready to be used by pharmacy students, health care practitioners and academics and will continue to be refined as required.

## Background

The use of Complementary and Alternative Medicine (CAM) has steadily increased in North America in the last 2 decades. Coinciding with the increase in use, the scientific evaluation of the efficacy and safety of CAM practices and products is also on the rise. In fact, a recent editorial reported that in 2008 more than 7500 CAM trials were indexed in MEDLINE [1].

The evaluation of the efficacy of CAM practices and products using the same methodology used for conventional practices and products is controversial. It can be argued that a randomized controlled trial (RCT), considered to be gold standard methodology in evaluating conventional medicine, may not adequately approximate how the CAM is used in practice and therefore may not be a fair way to assess efficacy. Of all the practices and products that make up CAM, natural products (NPs), including herbal medicines and natural sourced chemicals, most closely resemble conventional medications in terms of method of administration and the ability to quantify the administered dose. Like conventional medications, NPs are, therefore, well suited to evaluation using RCTs.

Health care professionals are accustomed to using evidence based information regarding the efficacy of conventional medicines as one part of their clinical decision making and therefore prefer to use evidence based information when helping patients make decisions about the use of CAM such as NPs. RCTs, conducted to determine efficacy of NPs, are being performed and published with increasing frequency. However, the critical appraisal of these trials, necessary to establish evidence based information, is more complex than with conventional medications due to the potential for variation in chemical content of NPs. RCTs completed using the best methodology do not reliably assess efficacy if the content of the NP being evaluated is not known or reported. The importance of the content of the NP and its potential for variation in content and thus activity is often not recognized or is underestimated. [1] This leads to difficulty in applying the results of RCTs of NPs to a patient's question about the utility of a particular NP.

The need to be able to assess the quality of RCTs of NPs prompted us to search the literature to identify an instrument to use in critically appraising RCTs of NPs. Our systematic review identified 16 published instruments for evaluating the quality of RCTs of NPs, however, none of the instruments stated that they had been validated and there did not appear to be a widely accepted gold standard instrument [2]. The CONSORT Statement for Herbal Interventions [3] came closest to fulfilling requirements for a quality instrument to critically appraise RCTs of NPs, however its content is specific for use with herbal medicines. There is therefore a need to develop an instrument that will have a broader use in critically assessing the quality of RCTs of NPs that include herbs as well as single, natural sourced chemicals. Initially we proposed identifying a critical appraisal instrument that was designed for evaluating RCTs of conventional medicines and then adding a section specific for NPs. We subsequently determined that although there are many instruments available, there did not appear to be agreement as to which instrument was the "gold standard".

The purpose of this project was to develop and evaluate a critical appraisal instrument that is sufficiently rigorous to be used in evaluating RCTs of conventional medicines, and also has a section specific for use with single entity NPs, including herbs and natural sourced chemicals.

## Methods

The development and evaluation of a critical appraisal instrument for evaluating RCTs of NPs was divided into 3 distinct phases: establishment of a list of items essential in describing the identify of an NP, establishment of a list of non-NP items important for evaluating the quality of an RCT and, finally, a pilot study to compare the new instrument to a published instrument for usefulness in evaluating the quality of RCTs of NPs and in helping to answer a clinical question.

### Phase 1. Establishment of a list of items essential in describing the identity of an NP

#### Selection of items

the initial list of items for this study was compiled from items contained in published critical appraisal instruments designed for RCTs of NPs as well as from items suggested by the research team.

#### Participants

A list of 14 potential participants from disciplines including: natural products chemistry/pharmacognosy (n = 6), botany (n = 1), ethnobotany (n = 2), pharmacology of natural products (n = 1) and critical appraisal (n = 4) was compiled. Each participant was assigned a unique code (known only to the research assistant) and was sent an individual recruitment letter via e-mail. In anticipation of willingness to participate, potential participants were also sent the initial list of NP items along with instructions for completion

#### Procedure

the Delphi process [4] was used to achieve consensus among a group of experts as to which items describing the identity of an NP were essential to consider when critically appraising an RCT of an NP. The consensus building process was conducted in 2 rounds using email. All email contact with participants was conducted through individual emails (not one email containing all 14 addresses) to maintain anonymity. The research assistant was responsible for receiving and collating all results, using participants' codes. All collated results viewed by participants had names and codes removed to maintain anonymity. Consensus was considered to have been reached when 80% of participants were in agreement with an item being designated as essential to include in the instrument.

#### Delphi Consensus-Round 1

participants who agreed to participate were instructed to review the list of NP items on the Round 1 study form and indicate, using "yes, no, or uncertain", whether they felt that each item was essential to include in an assessment instrument used to evaluate RCTs of NPs. Participants were encouraged to submit comments regarding the wording of items and suggestions for additional items they felt should be included. Round 1 study forms were to be completed and returned by e-mail within two weeks of receipt. A reminder e-mail was sent to participants who did not respond to the initial email request for participation or had agreed to participate and had not returned the completed Round 1 form.

#### Delphi Consensus-Round 2

Round 2 study forms were created by compiling all responses, comments and suggestions for addition, with all identifiers removed. Follow-up letters and Round 2 study forms were sent to each participant via individual e-mail. Participants were instructed to comment on the revised list of items as well as on any comments or suggestions recorded from Round 1. A reminder e-mail was sent to each participant who did not respond within 14 days.

#### Analysis

Round 1: Responses, comments and suggestions from all completed Round 1 study forms were recorded and reviewed, using participants' codes to maintain confidentiality. A revised list of items was compiled based on participants' feedback and investigator expertise and was used for Round 2. Round 2: Responses and comments on all completed Round 2 forms were recorded and analyzed and a final list of items considered to be essential by the study participants and investigators was assembled

### Phase 2. Establishment of list of important non-NP items

#### Identification of critical appraisal instruments for RCTs of conventional medications and NPs

A systematic review of the literature was conducted to identify critical appraisal instruments of RCTs of conventional medications and NPs, with the purpose of identifying items used in published instruments. Details from the portion of the systematic review pertaining to instruments designed for NPs were published previously [2]. Databases (PubMed, Embase, Web of Science, CINAHL, IPA and Cochrane Library) were searched from inception to June 2006 to identify potentially relevant articles that described the critical appraisal of RCTs of conventional medications. Search terms were: critical appraisal, scale, checklist, instrument, randomized controlled trial, systematic review, meta analysis, tool, form, evidence based medicine, quality control, quality assessment and standards. Searches were limited to articles written in English. Evidence-based medicine websites, textbooks and investigators files were searched for additional instruments. Bibliographies of relevant references were reviewed to identify additional articles.

Full articles of potentially relevant citations were retrieved and evaluated for relevance by two investigators, with disagreements being resolved by a third investigator. Articles were deemed relevant if they contained an instrument used for the assessment of RCTs of conventional medicine, described how to report an RCT of conventional medicine or discussed the importance of quality assessment and provided suggested items essential to the assessment. Articles were excluded if they contained an instrument that had been previously published, contained an instrument designed specifically for the assessment of a meta-analysis, a systematic review or for a trial design other than RCT or contained an instrument designed for the assessment of RCTs of therapies other than medication.

#### Compilation of items from critical appraisal instruments for conventional medications

a list of all items contained in assessment instruments for conventional medications and guidance documents was compiled, using a process similar to that used for the NP instruments [2]. A table, using the Revised CONSORT statement [5] as a framework, was used to record all items. Items contained in assessment instruments, but not found in the Revised CONSORT statement, were recorded in a separate section labeled as miscellaneous items. Items from instruments that were reported to have been validated and/or there was empirical evidence provided for the importance of including the items were noted.

#### Analysis

The list of all items from conventional instruments was examined for items essential for inclusion in the new critical appraisal instrument. To be designated as essential to include in the new critical appraisal instrument, an item had to meet at least 1 of the following 2 inclusion criteria: it had to have been contained in a published instrument that was documented as having been validated or must have had empirical evidence to support its inclusion in a published instrument. Originally we planned to have a third criterion, which was that the item had to have been included in the more than 50% of critical appraisal instruments. This criterion was removed from consideration for reasons that are provided in the Discussion section.

### Phase 3. Field test of new assessment instrument

#### Purpose

to evaluate the new assessment instrument by 1) comparing the performance of the new assessment instrument to that of a published assessment instrument in evaluating the quality of RCTs of NPs and helping to answer a clinical question; 2) measuring the validity of each criterion; 3) determining the potential impact of the order of use of the instruments.

#### Study design

the randomized trial was approved by the Dalhousie University Health Sciences and Humanities Human Research Ethics Board, with participants and primary researchers (TJ and AMW) being blinded as to which group the participants were assigned. To be eligible to take part in the study, participants had to fit one of the following inclusion criteria: be a practicing pharmacist, be a student currently enrolled in a pharmacy program or be an academic involved in the teaching of critical appraisal skills or clinical skills to pharmacy students. People were excluded from participating if they had been involved in any of the earlier phases of development of the new instrument. A sample size of 14 was determined to be sufficient, based on a similar study [6]. Participants were given 3 weeks to complete the study and return all materials. They received a reminder after 3 weeks if the completed material was not received by the research assistant and provided additional time, if required to complete the assessment.

#### Materials

Three published RCTs evaluating the efficacy of soy isoflavones in treating hot flashes were selected, based on having a range of quality of trials for the study. All identifiers, including authors, affiliations and journal citation were removed from the 3 articles. A clinical case describing a woman experiencing hot flashes as a consequence of menopause, was prepared. Participants were asked to apply the results of the RCT to the case by answering the following clinical question: "In a 53-year-old postmenopausal woman experiencing 5 – 10 hot flashes per day would soy isoflavone extract decrease the frequency and severity of the hot flashes?".

The draft new assessment instrument developed in the first 2 phases of this project, along with a User's Guide was provided for the appraisal of each RCT. A published assessment instrument used for reviewing clinical trials of herbal products was selected as a comparator instrument [7]. Permission was obtained from the publisher to use the assessment instrument. All identifiers were removed from both instruments to reduce the risk of bias.

#### Procedure

Participants were randomly assigned to 1 of 2 groups using a technique to conceal allocation that involved selecting 1 of 2 colors of paper out of a hat. Study packages each contained 2 envelopes, one red and one blue. Each envelope contained the clinical case and question, an assessment instrument, a User's Guide, 3 RCTs to be critically assessed and an evaluation form for feedback on the usefulness of the assessment instrument. All participants were instructed to open and complete the red envelope first. Half of the participants received red envelopes containing the new assessment instrument while the red envelopes received by the second group of participants contained the comparator instrument. Participants were told to open and complete the contents of the blue envelope only after completing the contents of the red envelope. After contents of both envelopes were completed, participants were instructed to fill out an evaluation form that asked which instrument they found easiest to use, which one was most helpful identifying strengths and weaknesses of the study and in answering the clinical question and which one was most helpful in selecting an NP comparable to one used in the RCT.

#### Analysis

all responses submitted for each item contained in the new critical appraisal instrument for each of the 3 articles were recorded by the research assistant (MM), using participants' codes rather than names to preserve anonymity. To test for criterion validity, Fisher's exact test was used to compare responses for each item submitted by each participant to the investigators "gold standard" answers that were reached by consensus prior to beginning the trial. Data obtained from participants' use of the comparator instrument was analyzed to determine if using the comparator instrument resulted in the same assignment of overall quality of each article as was found with the new appraisal instrument. Fisher's exact test was used to determine if the order in which the instruments were used affected responses to the new assessment instrument. Responses to questions on evaluation sheets were compiled. All comments on the evaluation sheets, including suggestions for improvement, were recorded and examined.

## Results

### Phase 1. Establishment of a list of items essential in describing the identity of an NP

#### Selection of items

Sixteen items describing an NP or its placebo were selected for inclusion in Round 1 study form (Table 1, column 1). Thirteen of the 16 items were found in 3 published instruments for NPs [3,7,8]. Item #16 (success of blinding) was included in one instrument and was added to the initial list [8]. Item #7 (lot number of product) and item # 15 (description of placebo) were not found in published instruments and were added by investigators.

**Table 1:** Results of Delphi Consensus-Round 1

*Item*	*Compiled * *Responses*			*Participant * *Comments*
	Yes	No	Un certain	
1. The genus of the test NP was stated.	9	-	2	• Items 1 & 2 assume an organism is used, but there are many chemical NPs, so we need to include an unambiguous name if it is a natural chemical e.g. arginine is ambiguous, L-arginine is correct.
2. The species of the test NP was stated.	9	-	1	• Need to be more specific by listing cultivars/varieties
3. The plant part used to prepare the test NP was stated.	10	-	1	• Also when and where harvested
4. How the test NP was processed/extracted was described.	9	1	-	• Chemistry and manufacturing information is required by the NP Regulations • Proprietary data but for government registration should be included but should not be reported in the publication
5. If test NP was a commercial product, brand name was stated.	8	1	1	• Interesting, but not essential. • Brand is important because of differences in manufacturing.
6. If test NP was a commercial product, the name of the manufacturer was stated.	9	1	-	
7. If test NP was a commercial product, the lot number was stated.	8	2	-	• Make sure they have enough supply for the complete trial and didn't use different lots throughout study
8. The name of important chemical(s)of the test NP was stated.	11	-	-	• The term "important chemicals" is vague • Full characterization or fingerprinting of the testing material • This could be a problem with some products as active agent(s) may not be known • Should have a percentage of the chemicals present listed • If standardized for the chemical content and the manufacturer wants to be public
9. The amount of important chemical(s) of the test NP was stated.	11	-	-	• The term "important chemicals" is vague • If the makers are not active compounds, there is no need to quantify each compound but it is always good to have some quantitative information
10. The test NP was analyzed for chemical content.	11	-	-	• For registration with to government yes but should be agreed upon by investigator and company before publication.
11. The dosage form of the test NP was stated.	10	-	-	
12. The dose of the test NP was stated.	10	-	-	
13. The frequency of administration of the test NP was stated.	10	-	-	
14. The route of administration of the test NP was stated.	10	-	-	
15. The placebo or comparison treatment and the test NP were matched in terms of taste, smell, appearance and dosing regimens.	10	-	-	
16. The success of blinding was evaluated.	10	-	-	

#### Participants

11 of 14 potential participants completed and returned the Round 1 study form. Follow-up letters and Round 2 study forms were e-mailed individually to 11 participants. Eight of 11 participants completed and returned the Round 2 study form. Specific reasons for not completing the study were not provided by dropouts.

#### Delphi Consensus-Round 1

contents of completed study forms were recorded (Table 1). In general, there was a significant amount of agreement with the list of items. In total, participants unanimously agreed on the inclusion of 9 of 16 items. The remaining 7 items each had 1 or 2 participants who either were uncertain or did not agree that the item was essential to include in the list. Participants offered suggestions for clarification of wording of some items. Several participants suggested the inclusion of additional items that they felt were missing from the list, such as the stability of the NP, whether the NP was prepared according to monograph specifications, if a certificate of analysis was provided, where and when the plant was harvested and details of the growing conditions.

#### Delphi Consensus-Round 2

**Figure 1:**
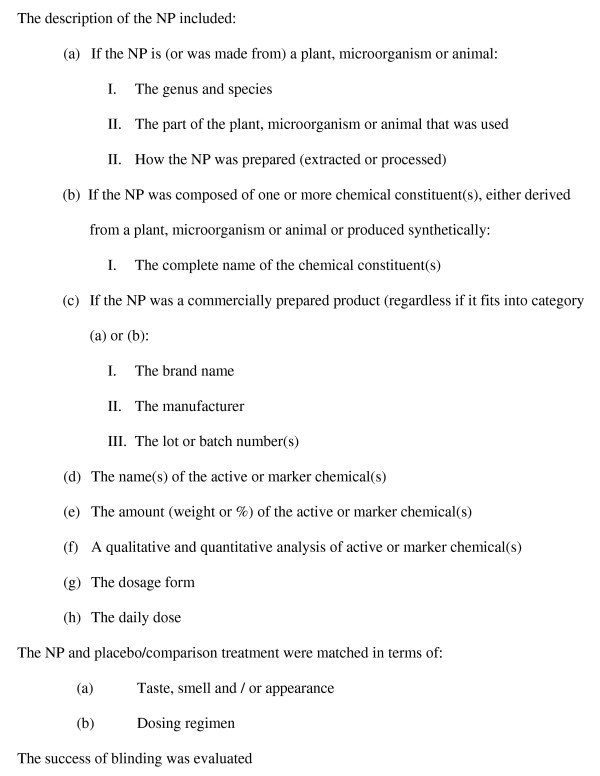
**Final list of items essential in describing the identity of an NP**.

the Round 2 form was created based on review of Round 1 results. There was at least 80% agreement on all items included on the Round 2 form. Thus these 15 items were considered by experts and investigators as essential. Twelve of the 15 items that pertained specifically to the description of the NP were grouped together (a-h) (Figure 1). Two other items were grouped together because they were specific to the matching of the NP and placebo/comparator.

### Phase 2. Establishment of important non-NP items

#### Identification of critical appraisal instruments for RCTs of conventional medications

**Figure 2:**
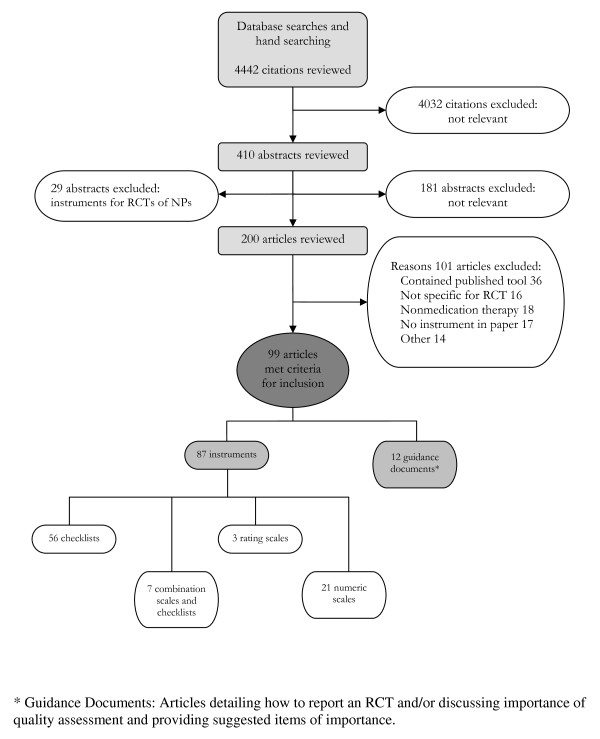
**Results of search for critical appraisal instruments for RCTs of conventional medications**.

The initial search of databases and hand searching of investigators files, websites, textbooks, and reference lists for critical appraisal instruments for RCTs of conventional medications and NPs identified 4442 citations (Figure 2). A review of the titles of the citations resulted in the exclusion of 4032 citations. Review of the remaining 410 abstracts identified 229 potentially relevant articles, 200 of which were for instruments specifically for RCTs of conventional medication. The remaining 29 potentially relevant articles pertained to instruments for RCTs of NPs and were systematically reviewed separately [2]. Upon further examination, 99 of the 200 articles met the inclusion criteria for instruments for RCTs of conventional medication [4,6,9-105]. The 99 articles consisted of 87 instruments for RCTs of conventional medications and 12 guidance documents written to guide reporting of an RCT and/or discussed the importance of quality assessment of RCTs of conventional medicine.

#### Compilation of items from critical appraisal instruments for conventional medications

4 of the 99 instruments stated that they had been validated [6,28,63,104]. Therefore, all 17 general item categories contained in the validated instruments were designated for inclusion in the new instrument (Table 2). Four of the 17 items categories: sequence generation (for randomization), blinding, participant flow and baseline data were further supported for inclusion by empirical evidence. [37,40,106-110]

**Table 2:** Support for inclusion of items in critical appraisal instruments for RCTs of conventional medicine.

**General Item Categories**	**Part of validated tool**	**Empirical evidence**
Participants	yes	
Interventions	Yes	
Objectives	Yes	
Outcomes (Methods)	Yes	
Sample size	Yes	
Sequence generation (Randomization)	Yes	Yes
Allocation concealment (Randomization)	Yes	
Blinding	Yes	Yes
Statistical flow	Yes	
Participant flow	Yes	Yes
Baseline data	Yes	Yes
Numbers analyzed	Yes	
Outcomes and estimation (Results)	Yes	
Ancillary analysis	Yes	
Adverse effects	Yes	
Interpretation	Yes	
Generalizability	Yes	

The new instrument for the critical appraisal of RCTs of NPs was assembled by combining the 17 general item categories identified as being important to assess with conventional medications (Table 2) with the essential items specific for the content of NPs determined in the Delphi consensus process (Figure 1). In some cases, item statements were further divided to provide clarity. For example, the general item category of "blinding" was divided into two items in the new assessment instrument: item #7. (The study was blinded) and item #24 (The success of blinding was evaluated). A section titled "reviewer's conclusion" was added to the end of the instrument to allow the reviewer to reflect on their findings after the evaluation of an article using the new instrument is complete. This included 2 questions to help the user apply the results to their practice. A User's Guide was developed to facilitate the correct and consistent use of the instrument. It included a section describing the purpose of the instrument and definition of each item

### Phase 3. Field test of new assessment instrument

Fourteen participants were enrolled in the RCT, pharmacists (n = 6), academics (n = 3) and pharmacy students (n = 5). Thirteen of 14 participants completed the study; 6 in the group that used the new assessment instrument first and 7 in the group that used the comparator instrument before using the new assessment instrument. The one dropout was unable to complete the study within the specified time frame due to unforeseen circumstances.

Responses, for each of the 3 articles, to each item in the new assessment instrument were recorded and compared between participants, regardless of the order in which the instrument was used. There was general agreement among participants as to the answers for most of the items in the new assessment instrument, for all 3 articles. Items producing the most discrepancy in answers were those dealing with the description of the NP.

Statistical analysis of participants' responses for each item in the new assessment instrument to the gold standard responses of the investigators for each of the 3 papers revealed that there was no significant difference in answers, indicating strong criterion validity Statistical analysis showed that the order in which the 2 instruments were used had no significant effect on responses to any of the items in the new assessment instrument.

The critical appraisal of the overall quality of each paper produced similar results, regardless of which instrument was used. That is, the paper that was judged to be of best quality of the three papers analyzed using the comparator instrument was also found by participants to be of the best quality using the new instrument

Participants' responses to the "Reviewer's Conclusion" section of the new assessment instrument were compared to the gold standard responses of the investigators. Eight of 13 participants (50% of those who used the new assessment instrument first and 71% of those who used the new assessment instrument second) felt that there was enough detail provided in Paper A (not in Paper B or C) to allow them to select a similar product, therefore agreeing with the gold standard. The group using the new assessment instrument second reported feeling much more comfortable in finding a product to recommend.

Participants who used the published assessment instrument first were fairly evenly split as to whether, after reviewing the papers, they would recommend the treatment to their patients. This was in contrast to those from the group who used the published assessment instrument after having used the new assessment instrument. They were more likely to say that they would not recommend the treatment.

The new assessment instrument was favored by participants in 3 out of 4 questions that participants completed as part of the Evaluation Form. Eleven of 13 found the new instrument to be most helpful identifying strengths and weaknesses of the studies, 8 of 13 felt that the new instrument was best at helping to answer the clinical question and 11 of 12 found the new instrument the most helpful in selecting an NP comparable to one used in the RCT. Participants were fairly evenly split as to which instrument they found easier to use. Overall, 12 of 13 participants preferred the new assessment instrument, stating that they liked the detailed questions about the identity of the NP. Feedback from the participant who preferred the comparator assessment instrument reported liking the numerical score that was generated by using the comparator instrument as an indication of quality. The new assessment instrument was favored over the published assessment instrument by the group who used the new instrument second, however, the participants who used the new assessment instrument first were less clear as to which instrument they preferred.

The new critical appraisal instrument was refined, based on participants' feedback and is included as Additional file 1. The final version of the User's Guide is presented in Additional file 2.

## Discussion

### Development of instrument

The Delphi process is a well recognized technique used to reach consensus among a group of experts [111]. Using content experts in the Delphi process to establish a list of essential NP items to include in the critical appraisal instrument ensured content validity of the items [111-113].

Results from Round 1 showed that participants, in general, agreed with the inclusion of the majority of the items in the new critical appraisal instrument. The depth and breadth of expertise of participants was evident in suggestions of additional items and changes in wording to improve clarity. The most challenging issue raised by participants in Round 1 was to identify the correct terminology to use for the chemicals contained in the NP. Items in the initial list asked for reports of RCTs to name "important" chemicals, their amounts and state whether the NP was analyzed for chemical content. Participants agreed that these items were essential but reported that the term "important chemicals" was vague or unclear. Assigning an unambiguous term to these chemicals is complicated by the fact that in some NPs, chemicals many have been determined to be "pharmacologically active", while in other NPs, in the active chemicals are unknown and so product content is standardized to marker chemicals.

Round 2 was used to achieve consensus on rewording of items and additional items as per suggestions from participants. The list of items was also reorganized for ease of use. Results of Round 2, coupled with investigators input, resulted in the list of essential items presented in Figure 1, which will become part of the new instrument for critical appraisal of RCTs of NPs. While the list contained many of the items found in other instruments, the Delphi consensus process, using the expertise of 11 participants, provided a forum for ensuring all items were essential to clearly define the NP being evaluated, were in language that was clear and unambiguous to users with a variety of backgrounds and were presented in a user friendly format.

Identification of non-NP items for the instrument was achieved by examining the content of 99 published instruments and guidance documents for the critical appraisal of conventional medications. Initially it was proposed that the items that appeared most frequently in the instruments would be likely to be the ones of most importance. While good in theory, there were two limitations with relying exclusively on frequency as an indicator of importance. Firstly, although there were 99 instruments and guidance documents to examine, the actual number of unique instruments was much smaller. This was a result of the fact that some instruments were not truly new instruments but were actually adaptations of earlier instruments. We felt that it was important to include these adapted instruments in our analysis as, the fact that researchers felt their initial content was worthwhile to retain and modify suggested their value. It was also important to identify what adaptations had been made as there was the possibility of identifying new items. The limitation of including these adapted instruments in our analysis was that there was the potential that some items could have been carried along into the revised instruments, increasing the frequency score of the item overall, but without the importance of the item being adequately assessed in the revision of the instrument. The second limitation of relying on frequency as the only indicator of an item's importance is that as understanding of critical appraisal develops, new items determined to be essential have been and will be added to new instruments. Because the items are new, their overall frequency of inclusion in instruments will be low and, by our criteria, would not have been included in our new instrument. It was for these two reasons that frequency of inclusion in an instrument was removed from the list of criteria for including non-NP items in our new instrument.

While frequency of inclusion of items in instruments is an indicator of their perceived importance, to overcome some of the inherent limitations described above, it was decided to include items that had empirical evidence to support their inclusion or were contained in instruments that said they had been validated. Only 4 of the 99 instruments were described by their authors as having been validated [6,28,63,104]. Interestingly, these 4 instruments were also designed using empirical methods of development including revisions based on inter-rater agreement or comments on content validity [6,63], Delphi consensus [28], and a modified Nominal Group Technique [104].

### Evaluation of instrument

The new assessment instrument, developed using a Delphi process and defined criteria to ensure that items had strong support for inclusion, was tested by anticipated users of a critical appraisal instrument. This included pharmacists, pharmacy educators and pharmacy students, each with a range of experience in critical appraisal and knowledge of issues important to NPs. An important part of the evaluation was to have participants be able to compare the new instrument to a published instrument that was designed specifically for use with NPs and that all identifiers from articles and instruments be removed to reduce the risk of bias.

Items in the new assessment instrument producing the most discrepancy in answers dealt with the identity of the NP. The range in answers was likely a result of lack of clarity in the items and so each of the NP items was examined and modified if required, to reduce ambiguity.

Randomizing the order in which participants used the 2 assessment instruments allowed us to probe whether the order affected their responses to items in the new assessment instrument. The fact that the order of use had no statistically significant affect on answers to items on the new assessment instrument allowed us to pool answers from both groups when assessing criterion validity by comparing participants' answers to the gold standard, thereby giving a larger sample size.

### Strengths and Limitations

The strength of the new assessment instrument described in this paper is the method used for its development. Identification and assessment of items for inclusion in the new assessment was based on clearly defined criteria. The use of experts in the Delphi process to guide the development of the list of items essential to include for NPs was clearly a strength, as was the evaluation of the instrument to validate criterion. This is an advantage over all published instruments for NPs that we identified.

A potential limitation of the instrument is that the initial list of both NP and non-NP items was dependent on the content of assessment instruments identified in the literature. The literature search was completed in June 2006 and it is possible, despite the fact that the investigators routinely monitor the newly published literature in this area, that newer instruments were not identified. It is also possible, due to the complexity of the nature of the database searching on this topic that not all relevant instruments were identified. Search results revealed that there was inconsistency in the key words used to index articles containing instruments and guidance documents. Both the time lag since the initial search and the difficulty in searching the databases could have resulted in some instruments not being identified thereby affecting the list of items. Additionally, because the search of the literature was limited to articles published in English, this may have also resulted in an incomplete identification of articles.

The number of items contained in the new instrument may be viewed as both a strength and a limitation. The number of items attests to its attempt to be complete in its assessment of the RCTs, however, some potential users may find the time needed to address each item prohibitive.

## Conclusion

A new instrument for the critical appraisal of RCTs of NPs was developed and evaluated. The NP section of the instrument was developed through consensus of a group of experts in NP and critical appraisal, ensuring content validity. The non-NP section of the instrument was constructed using items that were contained in instruments that had been validated or had empirical evidence to support their inclusion. The new assessment instrument was tested by a group of potential users who determined it to be helpful in evaluating RCTs to aid in answering a clinical question, as compared to a published comparator instrument. The instrument is distinct from other available assessment instruments for RCTs of NPs in its systematic development and validation. It has the additional advantage that its use is not restricted to herbs, rather it can be used with for all "single entity" NPs, such as glucosamine for example. The instrument is ready to be used by pharmacy students, health care practitioners and academics and will continue to be refined as required.

## Competing interests

The authors declare that they have no competing interests.

## Authors' contributions

TJ and AMW designed all studies, interpreted data and drafted manuscript. MMM conducted Delphi consensus rounds and the instrument evaluation trial. LL was responsible for database searching, relevance assessment and data extraction and some interpretation. All authors read and approved manuscript.

## Pre-publication history

The pre-publication history for this paper can be accessed here:



## Supplementary Material

Additional file 1 **Appendix A.** Final version of new assessment instrument developed for the critical appraisal of RCTs of NPs. Click here for file

Additional file 2 **Appendix B.** User's Guide for Dalhousie Assessment Instrument for Critical Appraisal of Randomized Controlled Trials (RCTs) of Natural Products (NPs). Click here for file
