# Supplementary material for: Development and evaluation of an instrument for the critical appraisal of randomized controlled trials of natural products
Source: BMC Complement Altern Med. 2009 Apr 23;9:11. doi: 10.1186/1472-6882-9-11 (PMC2687413; doi:10.1186/1472-6882-9-11)
Supplement: Additional file 1 — Appendix A. Final version of new assessment instrument developed for the critical appraisal of RCTs of NPs. [file 1472-6882-9-11-S1.doc]

Appendix A. Final version of new assessment instrument developed for the critical appraisal

of RCTs of NPs.

| **Dalhousie Assessment Instrument for Critical Appraisal of**  **Randomized Controlled Trials (RCTs) of Natural Products (NPs)** | | | | |
| --- | --- | --- | --- | --- |
| *Check the ‘Yes’, ‘Partially’, ‘No’ or ‘Unable to determine’ box beside each statement. Supplementary information explaining each item in more detail is located in the ‘Assessment Instrument - User’s Guide’ (e.g. definitions, examples, explanations, etc.).* | | | | |
| **Evaluation Statement** | **Yes** | **Partially** | **No** | **Unable to Determine** |
| **Introduction** | | | | |
| (1) The purpose of the study was clearly defined. | **□** | **□** | **□** | **□** |
| **Methods** | | | | |
| (2) The primary outcome of interest was clearly defined. | **□** | **□** | **□** | **□** |
| (3) The inclusion and exclusion criteria were clearly defined. | **□** | **□** | **□** | **□** |
| (4) The study was randomized. | **□** | **□** | **□** | **□** |
| (5) The process of randomization was described. | **□** | **□** | **□** | **□** |
| (6) The allocation (placement) of subjects into groups was concealed. | **□** | **□** | **□** | **□** |
| (7) The study was blinded. | **□** | **□** | **□** | **□** |
| (8) The natural health product (NP) under study was compared to:   1. Placebo and / or   (b) Accepted treatment | **□**  **□** | **□**  **□** | **□**  **□** | **□**  **□** |
| (9) The content of the placebo or comparison treatment was stated. | **□** | **□** | **□** | **□** |
| (10) The placebo/comparison treatment and NP under study were matched in terms of:  (a) Taste, smell and / or appearance  (b) Dosing regimen | **□**  **□** | **□**  **□** | **□**  **□** | **□**  **□** |

| **Evaluation Statement** | **Yes** | **Partially** | **No** | **Unable to Determine** |
| --- | --- | --- | --- | --- |
| (11) **If the NP is *plant, animal or microorganism*** based, **please answer items a – c**, skip d and then proceed to e.  **If the NP is a single *chemical***, please skip items a – c, **answer d** and then proceed to e*.*  The following information about the NP used in the study was provided: | | | | |
| (a) Genus and species  (b) Part of the plant, animal or microorganism used  (c) How NP was processed/extracted  (d) Full chemical name  (e) Brand name, if NP was a commercial product  (f) Name of manufacturer, if NP was a commercial product  (g) Lot number(s), if NP was a commercial product  (h) Name of active or marker chemical(s)  (i) Amount or percentage of active or marker chemical(s)  (j) If NP was analyzed for chemical content  (k) Dosage form  (l) Dose  (m) Frequency of administration  (n) Route of administration | **□**  **□**  **□**  **□**  **□**  **□**  **□**  **□**  **□**  **□**  **□**  **□**  **□**  **□** | **□**  **□**  **□**  **□**  **□**  **□**  **□**  **□**  **□**  **□**  **□**  **□**  **□**  **□** | **□**  **□**  **□**  **□**  **□**  **□**  **□**  **□**  **□**  **□**  **□**  **□**  **□**  **□** | **□**  **□**  **□**  **□**  **□**  **□**  **□**  **□**  **□**  **□**  **□**  **□**  **□**  **□** |
| (12) Methods to assess adherence were stated. | **□** | **□** | **□** | **□** |
| (13) A sample size calculation was performed to determine the minimum number of subjects needed for the study. | **□** | **□** | **□** | **□** |
| (14) The duration of the study was stated. | **□** | **□** | **□** | **□** |

| **Evaluation Statement** | **Yes** | **Partially** | **No** | **Unable to Determine** |
| --- | --- | --- | --- | --- |
| (15) The techniques and / or instruments used for measuring outcomes were clearly described. | **□** | **□** | **□** | **□** |
| (16) The methods of statistical analysis were clearly described. | **□** | **□** | **□** | **□** |
| **Results and discussion** | | | | |
| (17) The sample size was stated. | **□** | **□** | **□** | **□** |
| (18) Baseline characteristics were described and statistically analyzed. | **□** | **□** | **□** | **□** |
| (19) Regarding dropouts:  (a) Numbers were provided.  (b) Reasons were given. | **□**  **□** | **□**  **□** | **□**  **□** | **□**  **□** |
| (20) An intention-to-treat analysis was used. | **□** | **□** | **□** | **□** |
| (21) The results for each outcome were reported for all arms of the study. | **□** | **□** | **□** | **□** |
| (22) Statistical analysis was reported for each outcome for all arms of the study. | **□** | **□** | **□** | **□** |
| (23) P-values and / or confidence intervals were reported. | **□** | **□** | **□** | **□** |
| (24) The success of blinding was evaluated. | **□** | **□** | **□** | **□** |
| (25) The possibility of confounders was taken into account. | **□** | **□** | **□** | **□** |
| (26) Adverse effects were documented. | **□** | **□** | **□** | **□** |
| (27) The conclusion addresses the purpose of the study. | **□** | **□** | **□** | **□** |
| **reviewer’s conclusion** | | | | |
| (28) Based on my assessment of the article:   1. I feel that the paper was of sufficient quality to allow me to apply the results to my practice as one part of my decision making process 2. I feel that sufficient evidence was provided to allow me to select a specific product comparable to the NP used in the study. | **□**  **□** | **□**  **□** | **□**  **□** | **□**  **□** |

# 
