# Supplementary material for: Development and evaluation of an instrument for the critical appraisal of randomized controlled trials of natural products
Source: BMC Complement Altern Med. 2009 Apr 23;9:11. doi: 10.1186/1472-6882-9-11 (PMC2687413; doi:10.1186/1472-6882-9-11)
Supplement: Additional file 2 — Appendix B. User's Guide for Dalhousie Assessment Instrument for Critical Appraisal of Randomized Controlled Trials (RCTs) of Natural Products (NPs). [file 1472-6882-9-11-S2.doc]

# Appendix B. User’s Guide for Dalhousie Assessment Instrument for Critical Appraisal of Randomized Controlled Trials (RCTs) of Natural Products (NPs)

| User’s Guide for Dalhousie Assessment Instrument for Critical Appraisal of Randomized Controlled Trials (RCTs) of Natural Products (NPs)Developed by Drs. T Jurgens, Dr. AM Whelan and research team, College of Pharmacy,Dalhousie University, Halifax, NS, Canada. Dec 2008 |
| --- |
| The purpose of the ‘User’s Guide’ is to provide further explanation for items contained in the “Dalhousie Assessment Instrument for Critical Appraisal of Randomized Controlled Trials (RCTs) of Natural Products (NPs) |
| | (1) The purpose of the study was clearly defined. | | --- |   The objective was described thoroughly and specifically enough to determine if the trial was applicable to the reviewer’s practice (i.e. population, intervention, comparison, outcome). |
| | (2) The primary outcome of interest was clearly defined. | | --- |   The main outcome of interest to the investigators was stated.  Synonyms: endpoint, result |
| | (3) The inclusion and exclusion criteria were clearly defined. | | --- |   The list of characteristics that all potential participants must have had to be eligible to participate in the trial (e.g. inclusion criteria) was stated.  The list of characteristics that would have excluded potential participants from participating in the trial (e.g. exclusion criteria) was stated. |
| | (4) The study was randomized. | | --- |   The investigators stated that the trial was randomized.  Randomization is a method used to ensure that all participants entered in a trial have an equal chance of receiving each intervention. |
| | (5) The process of randomization was described. | | --- |   The method of randomization was clearly detailed so that it was likely that all participants had an equal chance of receiving each intervention.  Examples: (1) Random number tables; (2) Computer generated |
| | (6) The allocation (placement) of subjects into groups was concealed. | | --- |   The method used to ensure that the person(s) enrolling participants into the trial were unaware of the interventions to which the participants would be assigned, was described.  Examples: (1) Numbered or coded containers in which capsules from identical-looking, numbered bottles are administered sequentially; (2) On-site computer systems, where allocations are in a locked unreadable file; (3) Sequentially numbered opaque, sealed envelopes |
| | (7) The study was blinded. | | --- |   The investigators stated that the trial was blinded (e.g. the participants, clinicians, researchers, research assistants and/or data analysts were not aware of which intervention was being administered to each participant).  Synonym: masking |
| | (8) The natural product (NP) under study was compared to:  (a) placebo and/or  (b) accepted treatment | | --- |   The investigators stated that the NP was compared to either a placebo (inactive) or a treatment that is accepted/ recognized as being effective for the condition for which the NP is being investigated |
| | (9) The content of the placebo or comparison treatment was stated | | --- |   The composition of the placebo or the treatment to which the NP was compared, was stated. |
| | (10) The placebo/comparison treatment and NP under study were matched in terms of:  (a) taste, smell and / or appearance  (b) dosing regimen | | --- |  1. The investigators stated that the NP and the placebo/comparison treatment were similar with respect to taste, smell and/or appearance. 2. The investigators stated that the NP and the placebo/comparison treatment were similar with respect to their dosing regimen (i.e. frequency, route of administration, etc.). |
| | (11) If the NP is *plant, animal or microorganism* based, please answer items a-c, skip d and then proceed to e. If the NP is a single *chemical,* please skip items a-c, answer d, and then proceed to e. The following information about the NP used in the study was provided: | | --- |   Section (11) addresses the description of the NP. It is important that the identity of the NP used in the RCT be described in sufficient detail so that similar products may be selected for use in patients.  With the exception of single chemicals that are based on those found in nature, the majority of NPs are prepared from plants, microorganisms and animals. The chemical content (qualitative and quantitative) of a NP can vary significantly, depending on the species, plant part and method of preparation used. To make the results of the RCT applicable to all NPs with similar chemical content, it is essential that the investigators report details of the identity of the product being tested. This is true whether the NP was prepared from the natural source by the investigators or if a commercially prepared NP was used. |
| | (11) The following information about the NP used in the study was provided:   1. Genus and species | | --- |   The genus and species, the accepted botanical classification, were both stated. They are usually italicized, with the genus capitalized. Both the genus and species must be provided as a genus will often contain more than one species, each with its own chemical (and pharmacological) profile. Synonym: Latin binomial  Example: (1) *Echinacea purpurea* |
| | (11) The following information about the NP used in the study was provided:  (b) Part of the plant, animal or microorganism used | | --- |   The specific part(s) of the plant, microorganism or animal that was used to make the NP was stated.  Examples: (1) Root; (2) Aerial part   | (11) The following information about the NP used in the study was provided:  (c) How NP was processed/extracted | | --- |   The process used to prepare the NP was stated.  Examples: (1) The product was made by crushing the dried leaves and encapsulating the powder. (2) The tincture was made by extracting dried leaves with 50% aqueous ethanol.   | (11) The following information about the NP used in the study was provided:  (d) Full chemical name | | --- |   The name provided for the chemical constituent(s) was sufficiently detailed, including stereochemical designations, if appropriate, so that the identity of the chemical constituent(s) is clear and unambiguous. Examples: (1) “Glucosamine sulfate”, rather than “glucosamine”, to ensure that the salt form of the chemical constituent that was used in the trial is identified. (2) “Ginsenosides Rb1, Rg1, Rc, Rd”, instead of simply stating “ginsenosides”. |
| | (11) The following information about the NP used in the study was provided:  (e) Brand name, if NP was a commercial product | | --- |   The brand or trade name was stated. A similar response should be expected if the same brand of NP was used in a patient under similar circumstances. |
| | (11) The following information about the NP used in the study was provided:  (f) Name of manufacturer, if NP was a commercial product | | --- |   The company that produced the NP was stated. Many NPs are produced by several manufacturers. Identification of the manufacturer is a way to identify NP characteristics if the brand name is not unique.  Example: (1) Echinacea 100mg is the “brand name” on the label. Knowing the manufacturer would facilitate determining the source, content of the product. |
| | (11) The following information about the NP used in the study was provided:  (g) Lot number(s), if NP was a commercial product | | --- |   The specific lot or batch number of the product used was stated. This allows reviewers to determine if one lot or batch number was used to complete the trial. |
| | (11) The following information about the NP used in the study was provided:  (h) Name of active or marker chemical(s) | | --- |   The investigators listed compounds that have been identified in the product as being active or compounds that are known to be present in that particular genus and species (marker compounds). This is useful to help document that the correct plant has been used and can be useful when looking for a similar product to use. |
| | (11) The following information about the NP used in the study was provided:  (i) Amount or percentage of active or marker chemical(s) | | --- |   The quantity of the active or marker chemical(s), expressed as weight or percentage, was stated. The amount of each important chemical is helpful when trying to find a similar product. |
| | (11) The following information about the NP used in the study was provided:  (j) NP was analyzed for chemical content | | --- |   An analysis of the chemical content of the NP was conducted by the investigators or an independent laboratory on behalf of the investigators and results of the analysis were stated. Having the actual product that is used in the study analyzed for chemical content, rather than relying on label claims of the product, allows a better link between results of the study and chemical constituents.  Example: (1) The percentage of genistein was analyzed using chromatographic methods (HPLC, type of column and solvent). |
| | (11) The following information about the NP used in the study was provided:  (k) The dosage form | | --- |   The formulation used to administer the NP was stated.  Examples: (1) Capsule; (2) Tincture |
| | (11) The following information about the NP used in the study was provided:  (l) Dose | | --- |   The dose of the NP to be given in the trial stated. |
| | (11) The following information about the NP used in the study was provided:  (m) Frequency of administration | | --- |   The investigators stated how often the NP was taken.  Examples: (1) once daily; (2) twice a day; (3) once weekly |
| | (11) The following information about the NP used in the study was provided:  (n) Route of administration | | --- |   The investigators stated how the drug was taken by the study participants.  Examples: (1) orally, by mouth; (2) injection; (3) rectally |
| | (12) Methods to assess adherence were stated. | | --- |   The process that evaluated if participants adhered to the instructions for administering the NP and comparator was clearly described.  Synonym: compliance  Example: (1) The number of pills that remained at the end of the trial was stated. |
| | (13) A sample size calculation was performed to determine the minimum number of  subjects needed for the study | | --- |   The investigators stated that the minimum number of participants needed to achieve valid results was calculated prior to the start of the study. |
| | (14) The duration of the study was stated. | | --- |   The investigators described the length of the trial. |
| | (15) The techniques and /or instruments used for measuring outcomes were clearly  described. | | --- |   The investigators stated the means by which outcomes were measured. |
| | (16) The methods of statistical analysis were clearly described. | | --- |   The investigators stated the methods of statistical analysis used. |
| | (17) The sample size was stated. | | --- |   The total number of participants who participated in the trial was stated. Ideally, the total number enrolled, the number in each group and the number in each group who completed the trial, will be stated. |
| | (18) Baseline characteristics were described and statistically analyzed. | | --- |   The data obtained for each participant before the intervention was administered was stated. This usually includes demographics and clinical information. Additionally, statistical tests should have been conducted to determine how similar or different the groups were. |
| | (19) Regarding dropouts include:   1. Numbers were provided 2. Reasons were given | | --- |   The investigators stated the number of participants that did not complete the trial and the reasons why. If participants do not complete the trial (e.g. drop out), the exact number should be stated as well as the exact reason (e.g. adverse effects). |
| | (20) An intention-to-treat analysis was used. | | --- |   The investigators stated that an intention-to-treat analysis was used when analyzing the results. This is a method of analyzing the results that includes all participants that underwent randomization; participants are included even if they did not complete the trial. |
| | (21) The results for each outcome were reported for all arms of the study. | | --- |   The results from the measured outcomes were reported for all groups in the trial. |
| | (22) Statistical analysis was reported for each outcome for all arms of the study. | | --- |   The results for the outcomes of all groups were statistically analyzed and reported using the methods described in the ‘Methods’ section. |
| | (23) P-values and / or confidence intervals were reported. | | --- |   The level of statistical significance of the results was stated using p-values and/or confidence intervals.  The p-value is the probability (from 0 – 1) that the result(s) occurred by chance. The statistical significance increases as the p-value gets smaller.  Example: (1) A p-value of ≤0.05 is usually considered to be statistically significant; meaning that the observed results unlikely occurred solely by chance.  The confidence interval is the range of values that is believed to include the “true” value with high probability. A wider interval indicates less confidence in the results.  Example: (1) If the 95% confidence interval is (1.0, 2.0) that means that we can say with 95% confidence that the true value lies between 1.0 – 2.0. |
| | (24) The success of blinding was evaluated. | | --- |   The method used to measure if participants, researchers and/or data analysts remained unsure of what treatment the participants were receiving throughout the trial, was stated.  Example: (1) Patients were asked if they felt they had received the NP being studied or the comparator. |
| | (25) The possibility of confounders was taken into account. | | --- |   The investigators discussed any potential factor that is unrecognized or unmeasured that may affect conclusions that may be drawn regarding the relationship between a variable and an outcome.  Example: An investigator is trying to determine if consumption of alcohol is related to cancer and concludes from their study that regular consumption of alcohol does cause lung cancer. However, a closer look at the study population finds that the study participants were also more likely to smoke cigarettes. It is known that cigarette smoking causes lung cancer. So, in this case, cigarette smoking is the confounding factor. In other words, it is truly the smoking that causes lung cancer rather than alcohol. Potential confounding factors, such as this, must be taken into consideration when the trial is being designed. Otherwise, investigators may reach the wrong conclusions.^1^ |
| | (26) Adverse effects were documented. | | --- |   The undesired effects from the interventions were described. |
| | (27) The conclusion addresses the purpose of the trial. | | --- |   In the conclusion of the paper, the investigators related the results back to the original purpose of the trial. |
| | (28) Based on my assessment of the article:  (a) I feel that the paper was of sufficient quality to allow me to apply the results to  my practice as one part of my decision making process. | | --- |   This question addresses the goal of the assessment instrument. Overall, does the reviewer believe the conclusions of the study enough to use them in their own practice as one part of the decision making practice? E.g. taking into consideration the evidence that has been assessed, clinician experience and patient values. |
| | (28) Based on my assessment of the article:  (b) I feel that sufficient evidence was provided to allow me to select a specific  product comparable to the NP used in the study. | | --- |   The reviewer should determine if he/she feels that enough information about the content of the NP was provided in the description to allow them to find an NP of similar content. |

### Bibliography for User’s Guide

(1) Glossary: Definition of Nutrition and Medical Terms Used on This Website – Confounder. Cancer Nutrition Info. [http://web.cancernutritioninfo.com/glossary.cfm?id=36.](http://web.cancernutritioninfo.com/glossary.cfm?id=36.\) Accessed Feb. 8^th^, 2006.

(2) Ajetunmobi O. Making Sense of Critical Appraisal. London (GB): Hodder Headline Group; 2002.

(3) Barrett M, Editor. The Handbook of Clinically Tested Herbal Remedies. Volume 1. New York: The Hawthorn Press, Inc; 2004.

(4) Altman DG, Schulz KF, Moher D, Egger M, Davidoff F, Elbourne D, et al: The revised CONSORT statement to cluster randomized trials: explanation and elaboration. Ann Intern Med 2001; 134: 663-694.

(5) Glossary of EBM Terms. Centre for Evidence-Based Medicine, University Health Network. <http://www.cebm.utoronto.ca/glossary/>. Accessed Feb. 8^th^, 2006.

(6) Guyatt G, Rennie D, Editors. User’s Guide to the Medical Literature: A Manual for Evidence-Based Clinical Practice. American Medical Association; 2002.

(7) Glossary Index. Bandolier. <http://www.jr2.ox.ac.uk/bandolier/glossary.html>. Accessed Feb. 8^th^, 2006.

(8) Jadad AR, Moore RA, Carroll D, Jenkinson C, Reynolds DJ, Gavaghan DJ, et al. Assessing the quality of reports of randomized clinical trials: is blinding necessary? Control Clin Trials 1996; 17 (1): 1-12.
